# Supplementary material for: Genomic Signatures of Local Adaptation under High Gene Flow in Lumpfish—Implications for Broodstock Provenance Sourcing and Larval Production
Source: Genes (Basel). 2023 Sep 26;14(10):1870. doi: 10.3390/genes14101870 (PMC10606024; doi:10.3390/genes14101870)
Supplement: Supplementary file 1 [file genes-14-01870-s001.zip › genes-2596356-supplementary.pdf]

Supporting Materials for:

Genomic Signatures of Local Adaptation under High Gene Flow in Lumpfish—Implications for Broodstock Provenance Sourcing and Larval Production

Simo Njabulo Maduna [1,*], Ólöf Dóra Bartels Jónsdóttir [2], Albert Kjartan Dagbjartarson Imsland [2,3], Davíð Gíslason [4], Patrick Reynolds [5], Lauri Kapari [6], Thor Arne Hangstad [7], Kristian Meier [8] and Snorre B. Hagen [1]

1 Department of Ecosystems in the Barents Region, Svanhovd Research Station, Norwegian Institute of Bioeconomy Research, 9925 Svanvik, Norway; snorre.hagen@nibio.no
2 Akvaplan-Niva Iceland Office, Akralind 6, 201 Kópavogur, Iceland; odj@akvaplan.niva.no (Ó.D.B.J.); aki@akvaplan.niva.no (A.K.D.I.)
3 Department of Biological Sciences, High Technology Centre, University of Bergen, 5020 Bergen, Norway
4 Matís, Vínlandsleið 12, 113 Reykjavík, Iceland; davidg@matis.is
5 GIFAS AS, Gildeskål, 8140 Inndyr, Norway; pat.reynolds@gifas.no
6 Akvaplan-Niva, Framsenteret, 9296 Tromsø, Norway; lauri.kapari@akvaplan.niva.no
7 Lumarine AS, Stadionveien 21, 4632 Kristiansand, Norway; tah@lumarine.no
8 Lerøy Aurora, Stortorget 1D, 9008 Tromsø, Norway; kristian.meier@leroyaurora.no
* Correspondence: simo.maduna@nibio.no; Tel.: +47-902-89-810

Table of contents:

**Extended Materials and Methods**	Page 2
**Extended Results**	Page 3
**Figure S1**	Page 3
**Figure S2**	Page 4
**Figure S3**	Page 6
**Figure S4**	Page 6
**Figure S5**	Page 7
**Figure S6**	Page 8
**Table S1**	Page 9-10
**Table S2**	Page 11
**Supplementary References**	Page 11

Extended Materials and Methods

1. 3RAD library preparation and sequencing

We prepared RADseq libraries using the Adapterama III library preparation protocol of Bayona-Vásquez *et al*., (2019; their Supplemental File SI). For each sample, ~50-100 ng of genomic DNA were digested for 1 h at 37 °C in a solution with 1.5 µl of 10x Cutsmart® buffer, 0.25 µl (NEB®) of Read 1 enzyme (*MspI*) at 20 U/µl, 0.25 µl of Read 2 enzyme (*BamHI-HF*) at 20 U/µl, 0.25 µl of Read 1 adapter dimer-cutting enzyme (*ClaI*) at 20 U/ µl, 1 µl of i5Tru adapter at 2.5 µM, 1 µl of i7Tru adapter at 2.5 µM and 0.75 µl of dH_2O. After digestion/ligation, samples were pooled and cleaned with 1.2x Sera-Mag SpeedBeads (Fisher Scientiifc™) in a 1.2:1 (SpeedBeads:DNA) ratio, and we eluted cleaned DNA in 60 µL of TLE. An enrichment PCR of each sample was carried with 10 µl of 5x Kapa Long Range Buffer (Kapa Biosystems, Inc.), 0.25 µl of KAPA LongRange DNA Polymerase at 5 U/µl, 1.5 µl of dNTPs mix (10 mM each dNTP), 3.5 µl of $MgCl_2$ at 25 mM, 2.5 µl of iTru5 primer at 5 µM, 2.5 µl of iTru7 primer at 5 µM and 5 µl of pooled DNA. The i5 and i7 adapters ligated to each sample using a unique combination (2 i5 X 1 i7 indexes). The temperature conditions for PCR enrichment were 94 °C for 2 min of initial denaturation, followed by 10 cycles of 94 °C for 20 sec, 57 °C for 15 sec and 72° for 30 sec, and a final cycle of 72 °C for 5 min. The enriched samples were each cleaned and quantified with a Quantus™ Fluorometer. Cleaned, indexed and quantified library pools were pooled to equimolar concentrations and were sent to the Norwegian Sequencing Centre (NSC) for quality control and subsequent final size selection using a one-sided bead clean-up (0.7:1 ratio) to capture 550 bp +/- 10% fragments, and the final paired-end (PE) 150 bp sequencing on one lane of the Illumina HiSeq 4000 platform.

Extended Results

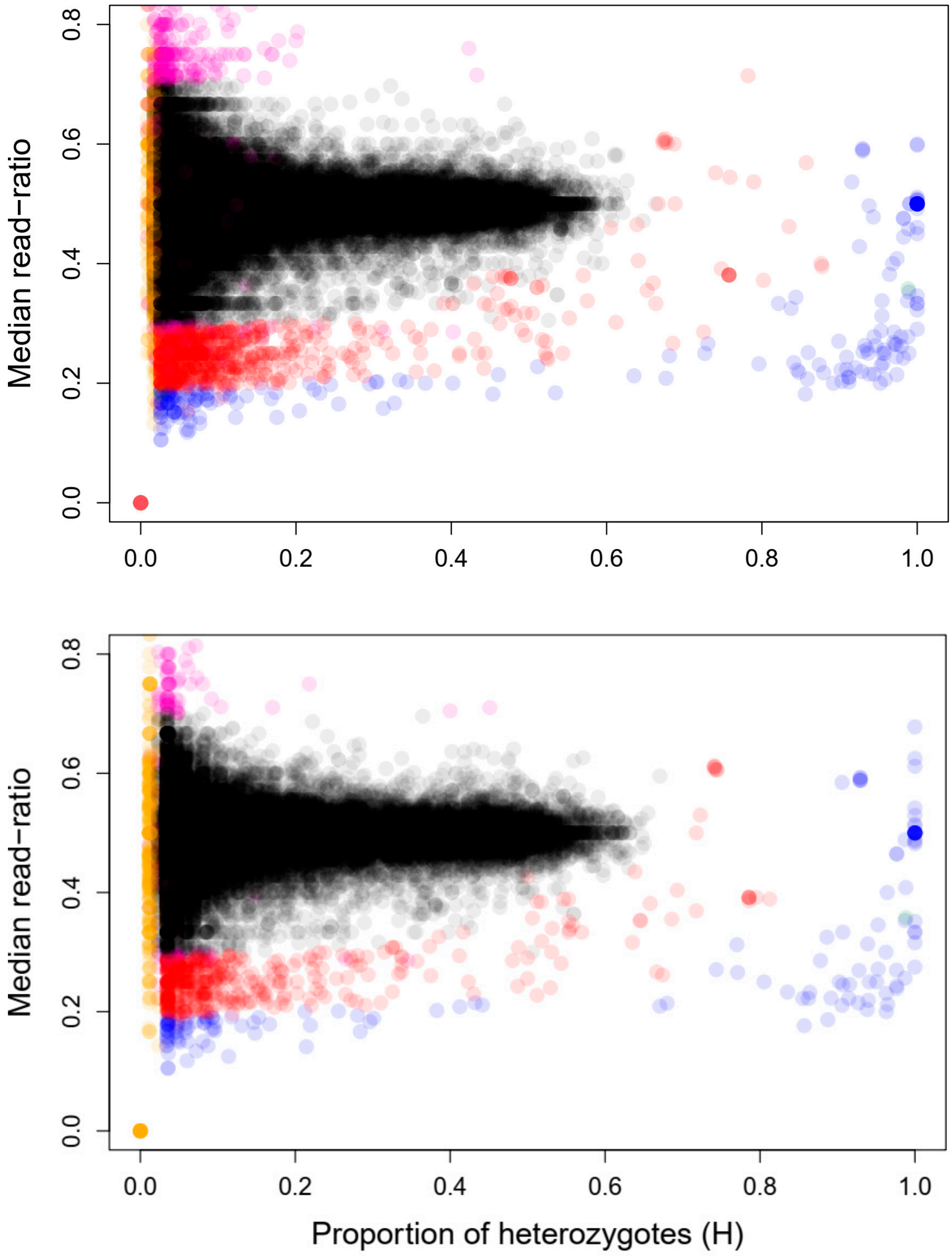

Figure S1. Characterization of duplication effect over the global (A) and regional (B) SNP datasets. The bivariate scatter plot display of the distribution of the SNPs with the median read-ratio deviation of heterozygotes (y-axis) plotted against the proportion of heterozygotes (x-axis). The median read ratio describes the deviation from equal alleles read ratio (50/50) expected for heterozygotes. Black, red, blue and orange points represent singletons, duplicated, diverged and low confidence SNPs, respectively.

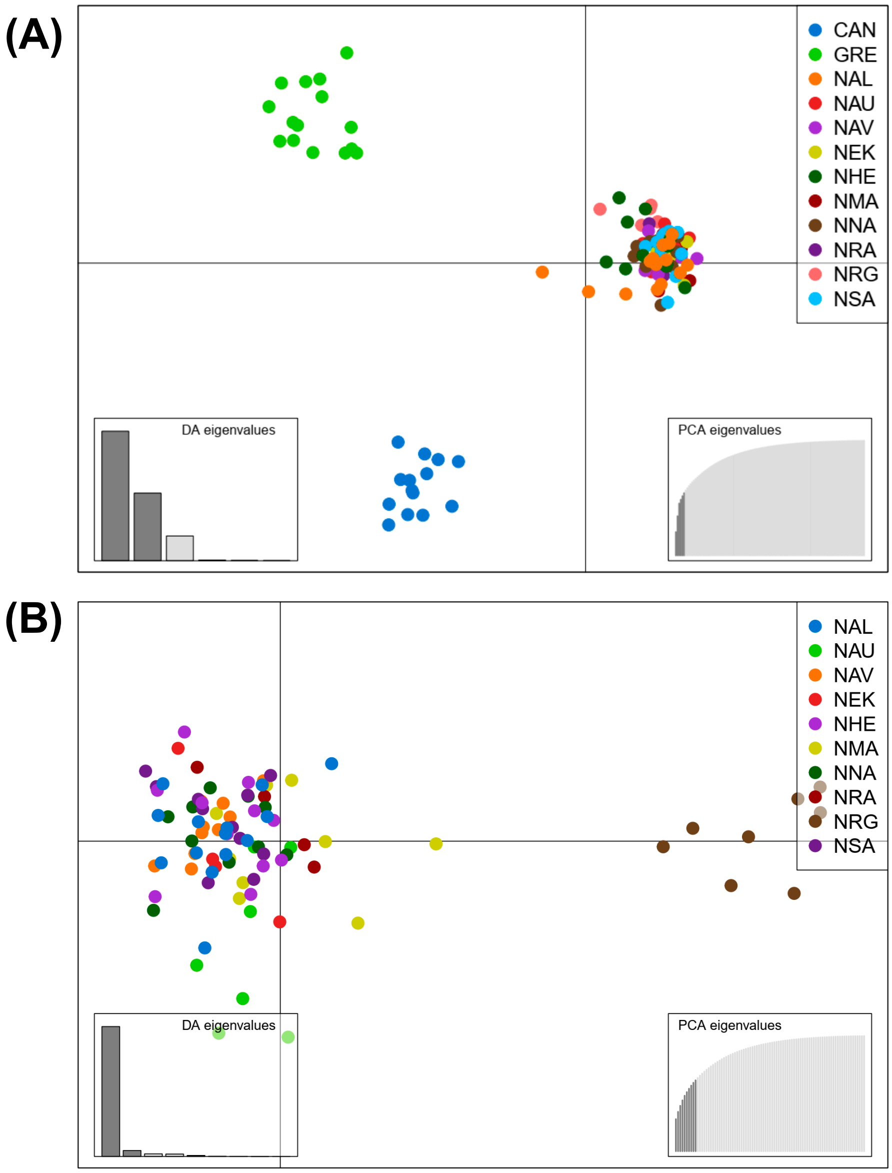

Figure S2. DAPC scatterplots showing adaptive variation based on candidate SNPs under positive (divergent) selection for the global (A) and regional (B) sampling scheme.

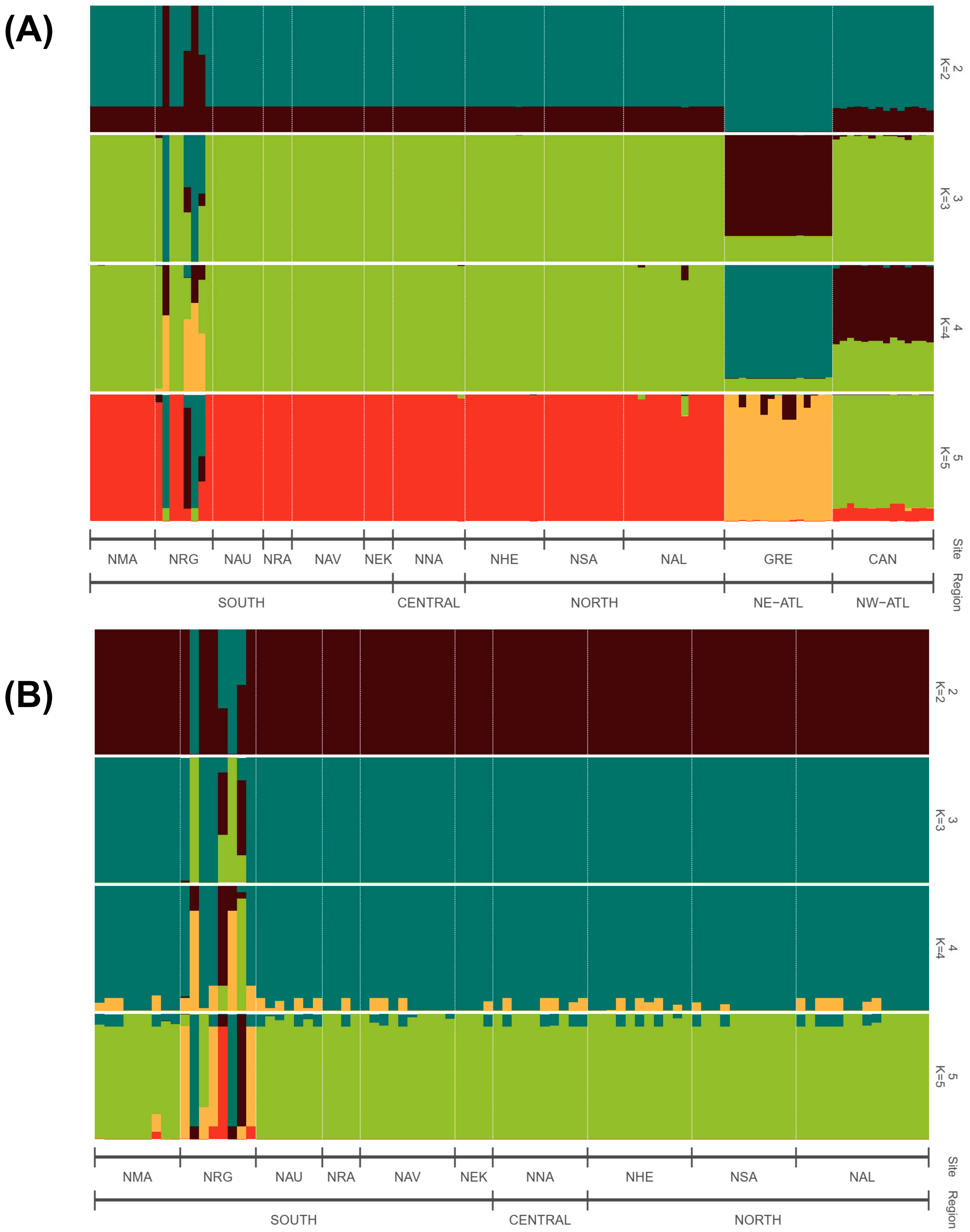

Figure S3. Sparse non-negative matrix factorisation (snmf) clustering analyses based on candidate SNPs under positive (divergent) selection for a global (A) and regional (B) picture of genetic cluster patterns of lumpfish across the north Atlantic for 10 independent runs of K = 2–5 based on the full neutral SNP dataset. A single vertical line represents each individual; a black line separates sample sites; the whole sample is divided into K colours representing the number of clusters inferred. The colours show the estimated individual proportions of cluster membership.

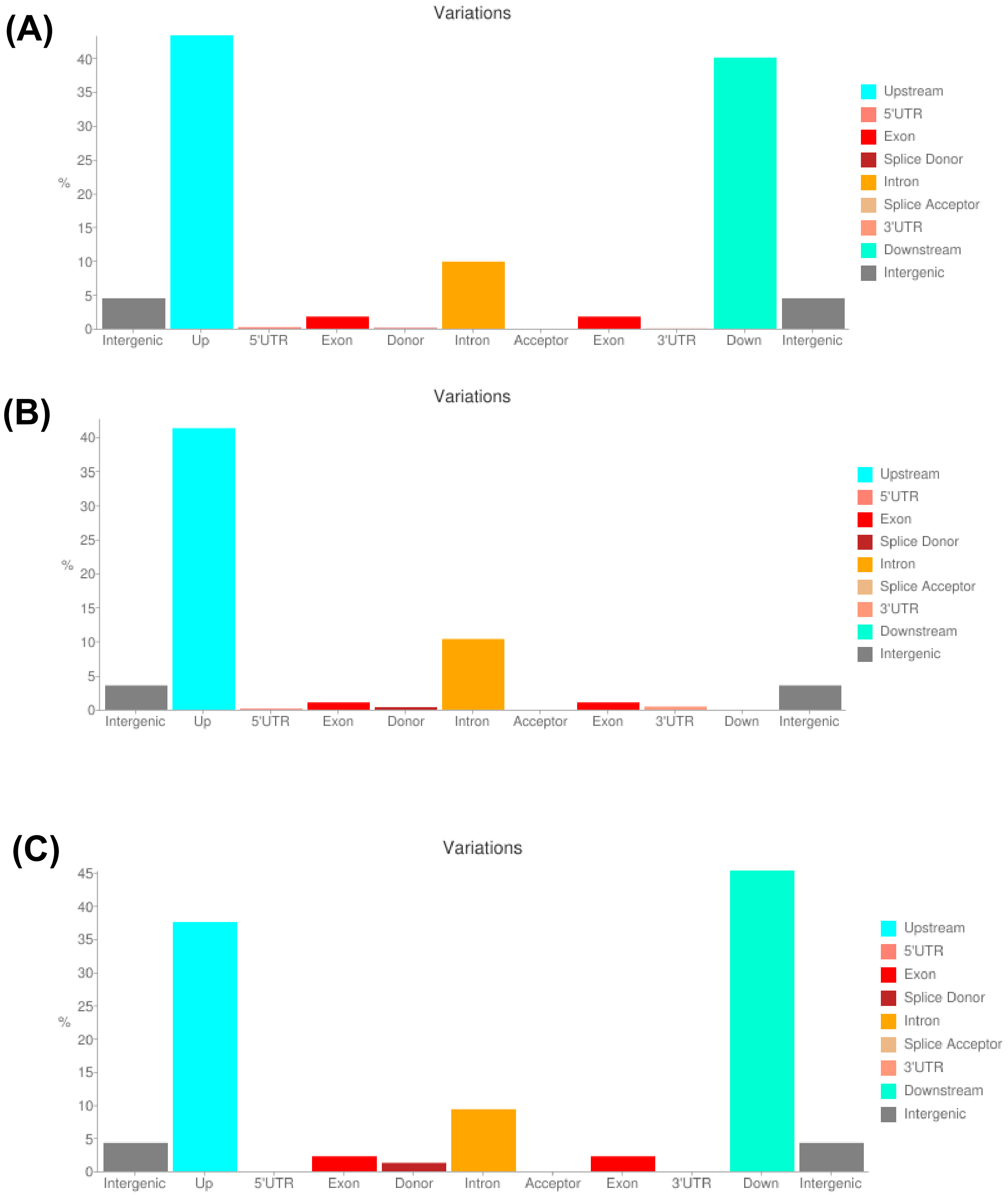

Figure S4. Annotation of environment-associated SNPs for the global (A) and regional (B) datasets. Overlapping between datasets are shown in (C).

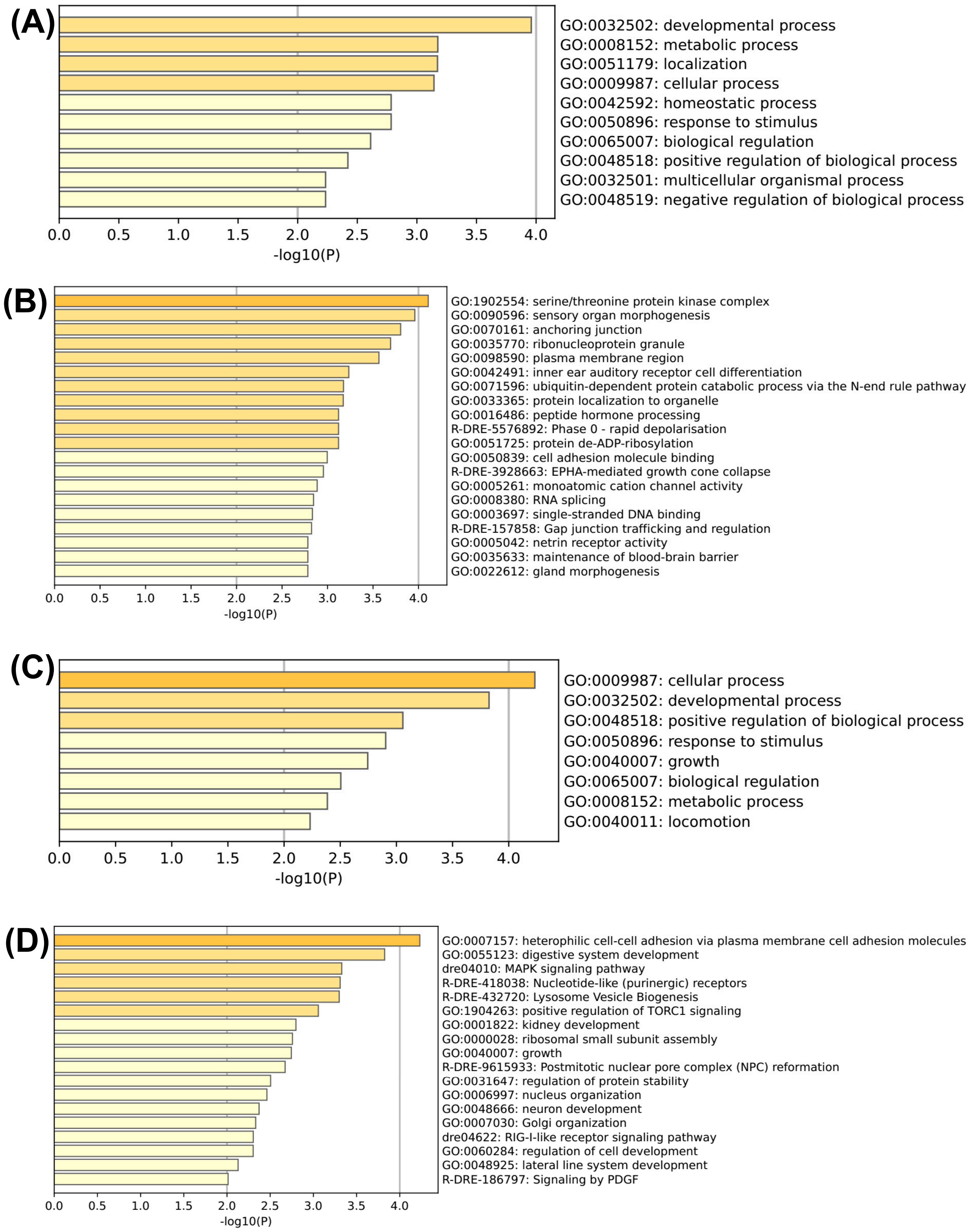

Figure S5. Functional annotation of environment-associated SNPs for the global (A & B) and regional (C & D) datasets. (A & C) The top-level Gene Ontology biological processes. (B & D) Top 20 clusters with their representative enriched terms (one per cluster), where "log10(P)" is the p-value in log base 10.

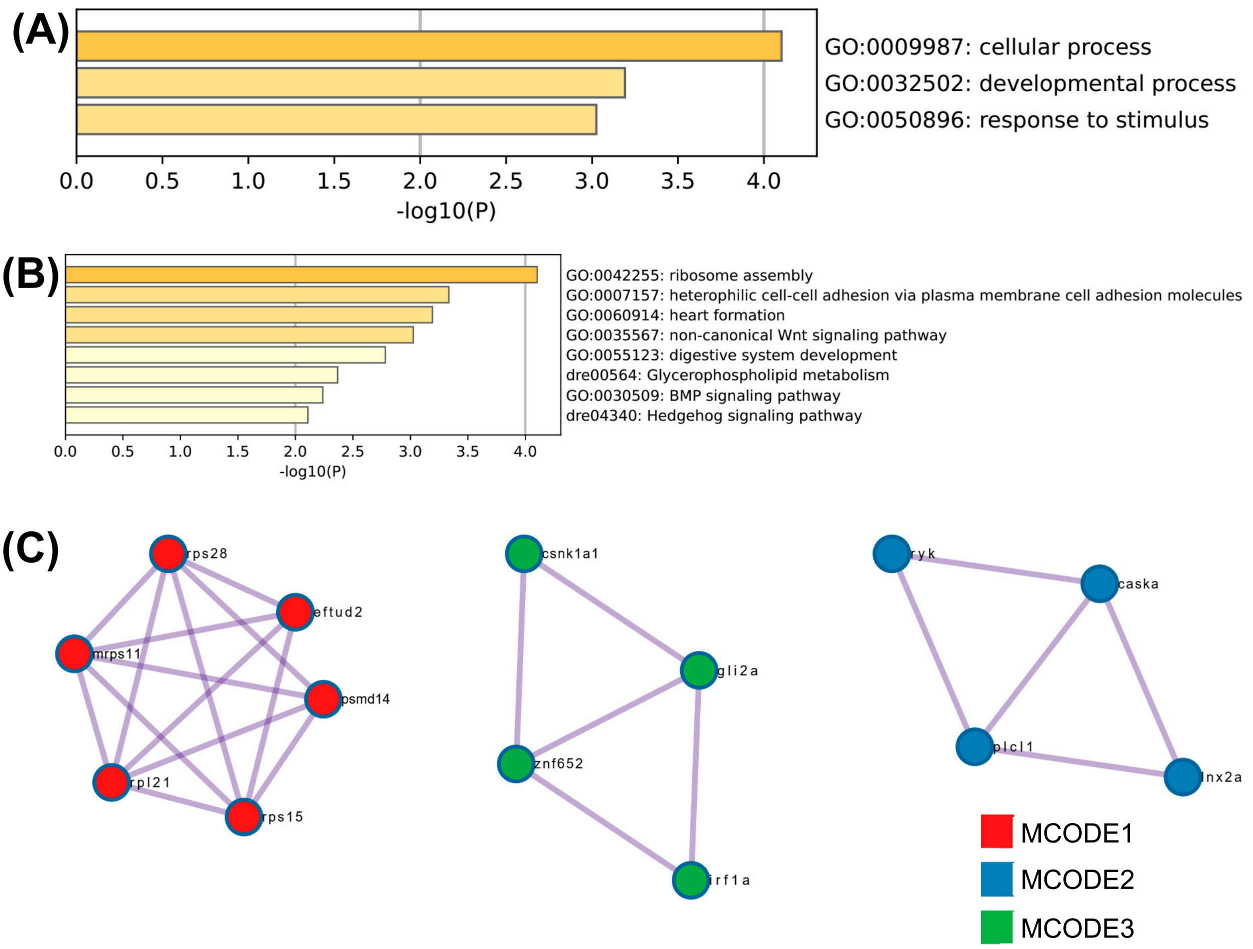

Figure S6. Functional annotation of 35 overlapping environment-associated SNPs between the global and regional datasets. (A) The top-level Gene Ontology biological processes. (B) Top 8 clusters with their representative enriched terms (one per cluster), where "log10(P)" is the p-value in log base 10. (C) Protein-protein Interaction Enrichment Analysis based on the Molecular Complex Detection (MCODE) algorithm to identify densely connected network components.

Table S1. Information of the environmental variable obtained from CMEMS*.

Product Name	Variable Name	Temporal Range	Temporal Resolution	Spatial Resolution	Derived Variables	Dataset Name
SST_GLO_SST_L4_REP_OBSERVATIONS_010_024	Sea Surface Temperature	1985-2014	Daily	0.05 °	OM, OMsd, HM, LM, HMsd, LMsd	ESACCI-GLO-SST-L4-REP-OBS-SST
GLOBAL_REANALYSIS_PHY_001_030	Sea Surface Salinity	1993-2014	Monthly	0.083 °	OM, OMsd	cmems_mod_glo_phy_my_0.083_P1M-m
	Sea Currents Velocity (Eastward)	1993-2014	Monthly	0.083 °	OM, OMsd	
	Sea Currents Velocity (Northward)	1993-2014	Monthly	0.083 °	OM, OMsd	
OCEANCOLOUR_GLO_BGC_L4_MY_009_104-TDS	Chlorophyll Concentration (CHLa)	1998-2014	Monthly		OM, OMsd	cmems_obs-oc_glo_bgc-plankton_my_l4-multi-4km_P1M
GLOBAL_MULTIYEAR_BGC_001_029	mass concentration of chlorophyll a in sea water (CHLm)	1993-2014	Monthly	0.25°	OM, OMsd	cmems_mod_glo_bgc_my_0.25_P1M-m
	mole concentration of phytoplankton expressed as carbon in sea water (PHYC)	1993-2014	Monthly	0.25°	OM, OMsd	
	mole concentration of dissolved molecular oxygen in sea water (O2)	1993-2014	Monthly	0.25°	OM, OMsd	
	mole concentration of nitrate in sea water (NO3)	1993-2014	Monthly	0.25°	OM, OMsd	
	mole concentration of phosphate in sea water (PO4)	1993-2014	Monthly	0.25°	OM, OMsd	
	mole concentration of silicate in sea water (SI)	1993-2014	Monthly	0.25°	OM, OMsd	
	mole concentration of dissolved iron in sea water (FE)	1993-2014	Monthly	0.25°	OM, OMsd	
	surface partial pressure of carbon dioxide in sea water (SPCO2)	1993-2014	Monthly	0.25°	OM, OMsd	
	sea water ph reported on total scale (PH)	1993-2014	Monthly	0.25°	OM, OMsd	
	net primary production of biomass expressed as carbon per unit volume in sea water (PP)	1993-2014	Monthly	0.25°	OM, OMsd	
OCEANCOLOUR_GLO_BGC_L4_MY_009_104	Suspended Matter (SPM)	1997-2014	Monthly	4 km	OM, OMsd	cmems_obs-oc_glo_bgc-transp_my_l4-multi-4km_P1M

Table S2 Environment-associated SNPs distribution across the lumpfish genome using the global and regional datasets. Overlapping number SNPs (N_{SNPs}) between datasets are defined as Common.

Chromosome	N_{SNPs}		
	Global	Regional	Common
1	37	8	3
2	12	1	1
3	37	11	4
4	28	4	3
5	27	3	1
6	21	2	-
7	26	2	-
8	19	4	3
9	29	5	
10	31	4	1
11	25	3	1
12	31	7	2
13	42	9	6
14	26	3	-
15	31	2	1
16	22	2	1
17	24	4	1
18	17	8	-
19	22	4	-
20	20	3	1
21	31	4	3
22	35	3	-
23	17	5	1
24	29	4	1
25	15	1	1
**Total**	**654**	**106**	**35**

2. Supplementary References

Bayona-Vásquez, N.J., Glenn, T.C., Kieran, T.J. et al. (2019). Adapterama III: Quadruple-indexed, double/triple-enzyme RADseq libraries (2RAD/3RAD). PeerJ, 7, e7724.
